# “Tell Someone,” to Both Women and Men

**DOI:** 10.3389/fpsyg.2021.673048

**Published:** 2021-05-10

**Authors:** Elena Duque, Patricia Melgar, Sara Gómez-Cuevas, Garazi López de Aguileta

**Affiliations:** ^1^Department of Theory and History of Education, University of Barcelona, Barcelona, Spain; ^2^Department of Education, University of Girona, Girona, Spain; ^3^Department of Sociology, University of Barcelona, Barcelona, Spain; ^4^Department of Curriculum and Instruction, University of Wisconsin–Madison, Madison, WI, United States

**Keywords:** new alternative masculinities, feminism, communicative acts, language of desire, violence against women

## Abstract

Contrary to an understanding of the struggle against gender violence as placing men and women in opposition to one another, victims have always been supported by both women and men. To prevent violence is important to know not only which message should be transmitted but also how the dialogue should unfold, and the characteristics of the people engaging in that dialogue. Because of the existing association between attraction and violence in our society, the unity of the language of ethics and the language of desire in such dialogue has become a key element in the struggle against gender violence. This study identifies the strong presence of communicative acts that unify these languages in the women (feminism) and men (New Alternative Masculinities) who are successful in this struggle. The opposition to violence that they defend guide their own desires, which are transmitted through their communicative acts to the people around them.

## Introduction

At the beginning of the investigation leading to this article, Rosa, a teacher who supervises recess, explained to one of the researchers her concerns about a situation that had recently arisen at her centre. A mother had contacted Rosa to tell her that the director of her daughter Nora’s school had called her, saying that in recent weeks a rumour had been going around the school that Nora had fellated Andrés, another student at the school. Andrés had been the one to initiate the rumour, giving numerous details about the incident and smearing Nora’s reputation. The faculty was most surprised when both students and professors had asked Nora about the situation, and she had confirmed it, proudly bragging. For her part, Nora commented that the student was now her boyfriend.

Rosa, the teacher who explained this situation to us, asked us the following question:

I would like to speak with Nora about this, to understand what’s going on, why she’s proud of this, what she thinks about the guy who is now her boyfriend, if it bothers her that he has chosen to spread private details of their intimacy and put her in a bad situation. However, there is one thing in particular that concerns me: how can I have this conversation in an effective way that will help her? I’m afraid to come off as moralising, imposing my criteria, so that she pushes back. It’s clear that the way we teachers have talked about these issues with the students until now has fallen on deaf ears: it seems like it hasn’t worked at all.

Violence against women has been studied in various disciplines, including sociology, psychology, law, and education, among others (De Koker et al., 2014; Stöckl et al., 2014; Ríos et al., 2018; Salceda et al., 2020). From this diversity of disciplines, there have been analyses of similarly diverse aspects of the reality surrounding the issue, i.e., its causes, profiles of victims and aggressors, circumstances surrounding aggressions, and preventive actions, among others (Melgar et al., 2021). However, there is an element that has been little studied and that corresponds to Rosa’s concern: how should all of this accumulated scientific knowledge be transmitted to the public so that people build relationships without violence? That is, which elements specific to communicative scenarios contribute to preventing or overcoming violence?

To prevent violence, however, it is important to know not only which message should be transmitted and how the dialogue should unfold but also who should speak during the dialogue. Is this an issue that only women should speak about, or should men be included? Will the inclusion of men itself effect a transformation?

Building upon previous scientific knowledge, in this article we first present a review of the current literature, which includes studies of dialogue, gender relations, and gender-based violence. In the second part of the article we present our study in which we use four everyday stories to demonstrate the specific elements of the communicative acts that are the key to generating desire in the construction of violence-free relations.

### Communication and Action for the Transformation of Gender Relations

Starting with the distinction between language-as-product and language-as-action (Clark, 1992; Trueswell and Tanenhaus, 2005), we focussed our study on the latter. Specifically, we focussed on how conversation constitutes a process of action-oriented collaboration. For this to occur, a certain understanding of the statements made is necessary, and this understanding of the statements is related to the identification of the speaker’s intention, that is, what each speaker intends by his or her expressions and what he or she is attempting to accomplish (Grice, 1957; Atkinson and Drew, 1979; Carston, 2002; Sperber and Wilson, 2002; Gibbs, 2003; Stone, 2005).

Searle (1969) emphasises the illocutionary force present in all speech acts; i.e., all speech acts contain elements that include the speaker’s intent. Thus, two identical propositional contents can contain different speaker intentions, which can be revealed through an analysis of their illocutionary force. Searle also identifies perlocutionary speech acts, such as those in which the propositional content does not explicitly contain the speaker’s intent, but does aim to have an effect on the listener. Soler and Flecha (2010) have later broadened these concepts, elaborating their theory of speech acts. These authors do not limit themselves to analysing speech acts; instead, they also examine other elements present in the communication, such as gestures, intonation, looks, and the context that surround the interaction. For Soler and Flecha (2010), these other components can determine whether a communication is a dialogic or a power interaction – that is, whether the listener is acting freely or under coercion. For example, an identical propositional content – “Shall we have a coffee?” – is different when it is produced in a context of leisure between two friends who have an equal relationship versus when is it produced in a work context and the speaker has a hierarchically superior position to the listener (for example, the speaker is the boss). Therefore, it is necessary to recognise the set of elements implied in the interaction – specifically, non-verbal communication (i.e., the social context in which the interaction occurs) (Glenberg and Kaschak, 2002; Stobbe, 2005; Klein, 2006), and tacit knowledge (Soler and Flecha, 2010). The latter is understood as the implicit knowledge that we take for granted; tacit knowledge conditions our beliefs, hopes, and fears and, consequently, the significance of what we intend to say. These components can condition not only comprehension of a message but also the actions triggered by that message.

### Construction of the Narrative about Violence Against Women

In the beginning, violence against women was conceptualised – i.e., it was narrated and interpreted – as an individual pathology derived from the aggressor’s problems with emotional management (specifically, anger) and the victim’s psychological problems, leading to masochistic tendencies, dysfunctional moral conceptions, etc. In the 1970s, anti-violence movements were born that opposed these conceptualisations and intended to generate an alternative, contra-narrative interpretation, of the reality of gender violence (Schechter, 1982; Dobash and Dobash, 1992). Thus, since the movement’s inception, gender violence has moved from being understood as an individual problem to being understood a social problem rooted in systems of patriarchy and gender inequality (Dobash and Dobash, 1992; Renzetti et al., 2001; Poore and Dabby-Chinoy, 2005; Lehrner and Allen, 2008). For this reason, with the goal of contributing to that counter-narrative, the principle focus of analysis has been to understand the elements that reinforce violence against women. Our discipline analyses the discourse to identify how violence is justified, how victims are blamed, or how the problem is minimised (Lloyd and Emery, 2000; Berns, 2001; Boonzaier, 2008; LeCouteur and Oxlad, 2011).

In this sense, these counter-narratives either confronted or deepened the rhetoric of men’s rights that justifies men’s violence (Adams et al., 1995), the moral discourses that surround domestic violence against women (LeCouteur and Oxlad, 2011), and the interpretations affirming that discourse is used to reproduce existing social order and power mechanisms (Foucault, 1978).

On occasion, this rhetoric or discourse has been reinforced by conglomerates of forms of speech that have created a normative language promoting discourse that has nurtured violence against women (Towns and Terry, 2014). One example is that of speech conglomerates composed of all of the words and expressions that either relegate women to an inferior position in the social hierarchy or render them invisible (King, 1991; Mills, 1995; Lillian, 2007; Coles and Fechter, 2012; Itakura, 2014). The first case has been well studied under the label of sexist language. The second case refers to the linguistic focus on the neutral gender, which for some authors implies a reformulation that marginalises both feminist voices and initiatives for social change (Riley, 2001; Palomares, 2009).

Considering the incidence of language in the social construction of subjects, the studies that centre their analysis of discourse tied to violence against women do not limit themselves to the content of the discourse. Instead, they also analyse the role of the speakers and more concretely, their gender identity.

If we conduct a historical retrospective, we find a confrontation that calls into question whether these discourses should be constructed and disseminated only by women, only by men, or by both together. More recent studies have set aside the superficial man-woman confrontation and underscore that what is important is to focus attention on the appropriate characteristics of men and women who construct discourses against gender violence (Gómez, 2015;; Puigvert, 2016; Puigvert et al., 2019).

Confrontation between the genders has been nourished by messages that have resulted in discrediting both women’s and men’s movements. For example, some messages present feminists as anti-equality activists, that is, feminists are characterised as seeking to recognise gender differences with the intention of generating unequal rights. Alternatively, some messages present feminists as extremists or crazies who exaggerate the historical oppression of women (Riley, 2001). With respect to men’s movements, some people’s views are premised on the conviction that antifeminism is the reason for their existence (Palma, 2008; Blais and Dupuis-Déri, 2012). These discourses, to the extent that they negate or minimise inequality and violence, make any transformative action seem unnecessary.

In recent years, this orientation has shifted and various sectors have recognised that the construction of movements fighting violence against women cannot be solely the responsibility of women: it is necessary to involve men (Crooks et al., 2007; Pinilla et al., 2014). This requires a change in the discourse, abandoning postures of blame and accusation that lead to all men being considered toxic agents and inviting men to a collaboration to advance the fight against gender-based violence from a place of respect (Joanpere and Morlà, 2019). Some programmes and campaigns, such as the Real Man Campaign, Green Dot, and Tell Someone, among others, have adopted this approach.^1^

However, just as we have advanced, the most recent studies on this issue take a further step and highlight the need to differentiate between those people who are part of the problem – those who either engage in some type of violence or are accomplices – and those who can be part of the solution if they join the discourse (Connell, 2000; Crooks et al., 2007; Gómez, 2015; Javaid, 2017; Shumka et al., 2017). One of the causes of the persistence of violence against women, not only between adults but also between young people (Ríos et al., 2019; Ruiz-Eugenio et al., 2020; Íñiguez-Berrozpe et al., 2021), is the socialisation of some people into certain models of attraction that unite desire and violence (Rebellon and Manasse, 2004; Castro and Mara, 2014). This socialisation is fostered through different interactions – family, friendships, the media, etc. (Elboj-Saso et al., 2020) – that reproduce a discourse in which people who manifest attitudes of domination and abuse attract others, whereas people who manifest egalitarian attitudes are presented as boring and even rendered invisible (Castro and Mara, 2014; Racionero-Plaza et al., 2021). This fosters attraction to people who are part of the problem, rendering invisible those who can contribute to overcoming it.

To encourage both men and women to contribute to the solution, we should pay special attention to ensuring that dialogues about affective and sexual relationships, or more generally about gender identities, join the language of ethics and desire (López de Aguileta et al., 2020), connecting attraction and desire with the values of friendship and solidarity (Rodríguez et al., 2014). Indeed, research has found that the discourses that promote the link between desire and violence separate the *language of desire*, referred to as “the capacity to raise attraction and be desired” (Flecha et al., 2013, p. 100) from *the language of ethics*, used to describe goodness and values. Breaking the union between desire and ethics promotes the double standards in which relationships in which there is domination are the exciting ones, whereas egalitarian relationships are seen as convenient but lacking pleasure (Torras-Gómez et al., 2020). However, language can be transformed through a re-socialisation process that enhances the awareness and critical reflection toward the link between attraction and violence, promoting rejection toward such model of attraction and propelling the union between desire and ethics in the same person (Gómez, 2015). In the case of men, we find this union in the identity that has recently been categorised as the New Alternative Masculinity (NAM), which is characterised by confidence, the courage to fight and ridicule of sexist and racist attitudes, an ethic that is combined with desire, uniting passion, friendship, solidarity, and equality (Portell and Pulido, 2012; Díez-Palomar and Mara, 2020). This union between ethics and desire is not only expressed and promoted through verbal language, but also through other communicative acts such as gestures, voice tone or gaze, among others, filling egalitarian attitudes with attractiveness and desire and emptying violent behaviours from any sort of attraction (Rodríguez et al., 2014; Racionero-Plaza et al., 2020). Taking communicative acts into account is therefore essential for overcoming gender violence (Flecha et al., 2020), as it is through the various elements of communication that attraction and desire are configured.

Therefore, to overcome the models of attraction tied to violence, it is not enough to identify violent attitudes or relationship, along with the prejudices they carry, nor is it enough to situate oneself exclusively in the language of ethics by simply counselling that this kind of relationship “isn’t good for you.” To overcome this kind of relationships requires knowing alternative models in a manner that combines the two languages when such models are transmitted and spoken about, for example by stating that relationships following this model not only are “good for you,” but also have “passion and desire” (Rodríguez et al., 2014).

Thus, in the study of gender-based violence and more specifically, in the analysis of the dialogues that generate transformative actions in affective and sexual relationships, deepening the understanding of how receivers identify the intentionality of speakers and how they understand the message is a matter of interest. It is necessary to observe which factors beyond propositional content affect listeners and lead listeners to join those discourses. These are the focus of our analysis in the next part in which we take as a reference the concept of communicative acts.

## Materials and Methods

### Communicative Methodology

In this study, we applied a communicative methodology (Gómez et al., 2011;, 2017; Gómez, 2019; Gómez et al., 2019; Redondo et al., 2020) to identify communicative acts that contribute to elaborating narratives that overcome violence against women. This methodology was successfully used in previous research funded and recognised by the European Commission (European Commission [Ec], 2010; Flecha and Soler, 2014). It has been used also successfully in specific research on communicative acts (Ríos and Christou, 2010; Portell and Pulido, 2012; Rodríguez et al., 2014; Ríos et al., 2018).

The analysis of the situation via communicative methodology is based on the idea that knowledge is the result of the dialogue between science and society. In ontological terms, reality is communicative, that is, it is a human construction in which the meanings are built through interactions (Redondo et al., 2020; Soler and Gómez, 2020). In epistemological terms, the evidence are the result of a dialogue based on the intersubjectivity (Stolorow et al., 1994; Gómez et al., 2006) of the participants in the research. The intersubjective perspective underlines that the interpretation of reality and generation of new knowledge are influenced by people’s meaning of reality (Flecha, 2000; Mercer, 2000).

This dialogue should confront both the arguments presented by the scientific community with respect to the issue studied and the arguments of the people participating in the study. The participants bring their lived experiences and interpretations from their *lifeworlds* (Schütz, 1967), which are contrasted with the scientific theories and investigations such that results represent the fruits of these interactions (Puigvert, 2014). Therefore, great importance is placed not only on listening to and collecting the opinions of the participants but also on working with the participants to interpret their contexts and social world.

### Sample and Data Collection Techniques

In this study, four everyday stories were told by key informants, who were the subjects of our interest because they have either personal or professional trajectories that tie them to the fight against gender-based violence.

Data collection that applies qualitative techniques of communicative orientation places special emphasis and care not only on the relationship that should be established between the researcher and the person being studied but also on the conditions necessary to favour dialogue. Therefore, throughout our investigation, the researcher established an egalitarian dialogue both in the collection process and in the interpretation of the results, empowering a relationship in which claims of validity – not claims of power – prevailed. In other words, we attempted to achieve a maximum reduction in the inequality that is often established between researchers (i.e., “experts”) and the people that they studied.

Throughout the stories, together with the storytellers, we analysed the communicative acts present in various conversations that they had had about gender-based violence. In telling the stories, the participants created a narrative reflection about conversations that they had either had or heard over the course of their lives about gender-based violence. The intent was not to reconstruct a biography but instead to establish a joint dialogue with their reality and to interpret how their thoughts are configured in the present.

The script used by the investigator aimed to obtain information about the differences between those communicative acts that have influenced current narratives and that they consider as contributing to overcoming violence, on the one hand, and those that do not make such a contribution, on the other hand. The elements present in the communication that were analysed in the interviews were not the content of the messages transmitted by the participants, but in the rest of the elements present in the communication, which are collected in the concept of communicative acts (Ríos et al., 2018). These elements were the tone of voice, looks, attitudes, gestures or any interaction that would have contributed to bring closer (or move away) or influence (or not) the people with whom they were conversing, regarding overcoming gender-based violence.

Following a purposeful sampling (Creswell, 2012) researchers intentionally selected individuals to understand the central phenomenon of the research, the communicative acts, providing useful information to go in depth. Criteria included for selection were a) having information that is relevant for the research due to their social movements implication or their professional; b) having the same number of men and women and c) being more than 18 years old. The study sample was composed of two women and two men.

Below, we present their profiles, highlighting elements that are pertinent to the study:

- Juan, 39-year-old man and factory worker. Three years ago he decided to join a men’s group; one of that group’s objectives was to overcome gender-based violence. Juan had not previously been active in other social movements. His participation in the association was motivated by a friend who was a member. It is important to note that although Juan had not previously been a member of other movements, at times he had attended debates and lectures about the topic of gender-based violence, although in general, those events had not awakened his interest.
- Daniela, 41-year-old secondary school teacher. For the last 2 years, she has been working on overcoming abusive relationships both in tutorials and in the framework of her classes. She does this by opening spaces for debate and, most significantly, through paedagogical discussion groups about scientific texts on the topic. During this time, Daniela has also attempted to learn more about these topics by attending lectures and reading articles. She has never participated in a women’s movement.
- Luis, 37-year-old secondary school teacher. Luis was involved in one of Spain’s first movements of egalitarian men, which he left after 2 years, feeling that the discourses promoted by the movement came from a concept of equality centred on the equitable distribution of domestic chores or the type of language used, which made him feel that they were far from his reality. Currently, he continues to defend the rights of women but he is not active in any movement. In his daily work at the school, he is very aware of gender issues – specifically, violence against women. Whenever possible, he attempts to discuss the issue with his students and even helps them attend lectures and workshops.
- Cristina, recess-supervising teacher who does not participate in any women’s movement or any movement against gender-based violence. Cristina has friends who are active in women’s and anti-violence movements, which keeps her sufficiently informed about the discourses promoted in such movements. In 2009, encouraged by one of these friends, she attended the state feminist conferences, and since then, she has regularly attended other lectures about feminism, lectures about masculinity, workshops against violence, and other classes related to gender in general. Simultaneously, she attempts to work on these issues with adolescents at her job whenever she has the opportunity.

Researchers collected the data through open-ended interviews with the participants. Once the information was collected, the researchers transcribed the data and conducted a *hand analysis* (Creswell, 2012). Researchers read several times the data to have a general sense of the content and to identify the communicative acts influencing gender violence. Finally specific categories were identified and presented in this article structured in 3 sections. According to the communicative orientation focussed on social transformation, the results reported are related to the dialogues that promote overcoming gender violence.

## Results

The results of this article collect the analysis of four people who participated in debates, workshops, conferences, and conversations with friends that had the principal theme of gender-based violence. The four people who participated in the fieldwork have heard a wide variety of discourses about this issue, and they came together to differentiate between a type of discourse that connects and helps generate transformation and a type of discourse that generates dismissal. Below, we present our results, which refer to the communicative acts present in dialogues that can influence the recipients of their messages.

### Women and Men Unifying the Language of Ethics and the Language of Desire

The four people who participated in analysing the communicative acts of men and women in the context of conferences, workshops, and informal conversations highlight coherence as a key element. Coherence means that when speaking about alternative models – in which there is no violence – communicative acts should attempt to unite the language of desire and the language of ethics. Simultaneously, when transmitting their words, the speakers should indicate that they are engaging in a real discourse that they themselves practice (or believe that they practice). This coherence is perceived, for example, via the non-verbal language present in the interactions either between speakers or with the rest of the participants.

Juan first refers to talks in which the speakers were all men, discussing how they interacted with each other. Second, Juan refers to his observations of some of his female friends who are active in women’s movements.

Juan: One of the speakers who was active in a pro equality masculinity movement, his whole discourse was “I am on top, I know what I’m doing.” However, **he transmitted, “Do what I say, not what I do.”** This is a pre-written discourse, but honestly, if you look at his face. you think “this guy has a double life, not even you believe what you’re saying” **He transmits an insecurity or a sense of “I think this, but I’m a little guy, I’m not worth anything.” I especially saw this in the way he treated the other speaker** – who obviously did have an egalitarian attitude – **he cut him off, he tried to make fun of him, he was going to like pound him. (.)**

**I pay more attention to someone who, when he’s speaking to me, transmits confidence and believes in what he does, rather than someone who doesn’t do what he’s defending.**

I have also been able to see this in female friends who are active in women’s movements and who call themselves radical. They generalise that all of us men are the same. However, the worst is that many of them end up hooking up with pricks. They are claiming that they are radical, but they don’t look at the nice guy, instead they focus on the biggest prick. So what they are reproducing, when they generalise, isn’t reality, but it’s their own experience, what they desire.

Daniela speaks to us about a lived example involving the students of the secondary school where she works. In 2014, she joined the students at a workshop for the prevention of gender-based violence. There were also people from other schools and of different ages at the event. Daniela’s words collect not only her own impressions but also those transmitted by her students after the session ended.

Daniela: When we went to see a conference where there was a feminist girl and a New Alternative Masculinities (NAM),^2^ they [the female students] still made a strong connection between the egalitarian man and the boring one, the ugly one. Seeing the speaker was attractive they said, “Ah, but if a NAM can be like that I like him too.” And I think that was **one of the things that helped them identify the NAM with a person who can be fun, attractive.** They also commented on **the good vibes, the complicity generated between him and her that they transmitted in general**. The fact that my students participated so much was a consequence of **the good vibe that they knew how to create, where it could be seen that between them there was an equal relationship.** **The confidence of the speakers permeated the air, and my students felt confident in speaking**, it seemed.

The collected examples discuss the bad praxis of speakers who talk about equality but treat others unequally. For example, they question disrespectfully or show more interest in people who are antagonistic to an egalitarian model. In the example given by Juan, we see how the speaker, through his or her interaction with other people, can reveal whether his or her acts correspond with what he or she is saying. In Juan’s example, the speaker looks down on and cuts off the other speaker, which completely undoes the entire theory that he is explaining. With respect to Juan’s female friends, we see that in the case of gender-based violence, coherence requires a union of ethics and desire. This means that the values and attitudes of people who are desired should correspond to the values and attitudes that are defended by the social movement. Therefore, defending equality means not choosing people who have attitudes that are violent, dominating, or abusive.

Conversely, the participants view as good those praxes in which the recipients have perceived that the message was not a learned utopic discourse that is impossible to put into practice. Instead, the speakers represented a way of relating that manifested a profound mutual respect, admiration, etc.

True influence is not achieved solely through ethical language – for example, saying, “this isn’t good for you” or “this person is dangerous for you.” Instead, as we have noted, influence also requires the language of desire. This second language can influence adolescents when the desire for an egalitarian person goes beyond theoretical discourse. Examples include when a look or tone of voice directed toward the egalitarian person transmits that desire, or when adolescents see people truly enjoying and feeling passion in egalitarian relationships.

### Having the Confidence and Bravery to Defend One’s Values and Oppose Violence

The stories related to coherence between theory and practice have also highlighted that coherence is transmitted through confidence. Specifically, when a message is being emitted, a look and a tone of voice must corroborate that the speaker believes in the truth of the message that he or she is transmitting. Daniela remembers an incident that occurred in one of her classes. She had been working with this group on the different models of attraction and, more specifically, on models connected to violence versus models of NAM. The contents of these models had been extensively analysed and debated. Daniela had already begun to note the transformations that these discourses had created amongst her students. The following example shows how these discourses positively influenced one of her female students and, specifically, how this student managed to transmit her attitude to the rest of the students.

Daniela: I remember a conversation between one girl and one of the boys, who had had a very aggressive reaction to me (the teacher): he raised his hand to me, he left the class. The next day, we were having an assembly, and she told him that: “now she knew that she would never go out with a person like him” And he asked her why and she told him, “because I know what will happen to me with a person like you.” The majority of the boys in the class supported this girl and disapproved of the other boy’s violent attitude.

She looked him in the eye without any fear while speaking to him. The level of confidence that she transmitted when speaking, the tranquillity, really surprised me. She didn’t get nervous at all; at least she didn’t let it show.

Confidence also brings with it the courage to defend equality and to position oneself against violence. In the speeches and workshops, confidence entails presenting the discourse without doubting or allowing oneself to be questioned, or in public spaces, like in Daniela’s class, publicly rejecting people who use violence and defending those people’s victims.

Thus, bravery is an attitude present in the transmission of discourses that effect transformation. This bravery is present in both the conception of a movement based on equality and the collective fight against gender-based violence in which the four participants coincide. The participants also note that in these movements, communicative acts that transmit inequality are unacceptable. In this case, we collect examples provided by Cristina, Luis, and Juan. Cristina’s example is from a government-led feminist conference in which most of the women present were active in a feminist association. This was not the case for Cristina, who was invited by a friend who was part of an association. These conferences touched upon very diverse topics, but one thing that really surprised Cristina was the tension that was tangible in the different debates and especially, the disregard with which some women spoke of men in general.

Cristina: Those who oppose men lose credibility when they oppose everything. **Everything they said was from this aggressiveness, rage, with this attitude that we’re going to destroy everything and that takes away all of their attractiveness and credibility.** What I noted there was that women are good and men are the opponents, and that’s it. Facing that, those of us who were listening were asking ourselves, What’s the point? To have women stick together so we get rid of all of the men, or to analyse what’s going on and build something with the reality that we have, which is men and women?

**Part of the key to making the other discourse reach farther is that it comes from communal work.** It’s not realistic for everyone to stick to his or her own side when increasingly things tend to be more egalitarian.

Juan had not previously been active in men’s movements, but in his story, he differentiates very clearly what, in his words, “gets him hooked” and what doesn’t.

Juan: For me, what some men have **transmitted in their talks. is that they promoted inequality.** If you tell a boy that what he needs to do is ask his partner forgiveness for everything that man has ever done to woman during all of humanity, always putting himself below. **honestly, it’s not attractive.**

**In the alternative, the first thing you see is respect,** and even if there’s some type of discrepancy, **people speak to each other with respect** with the idea that all of us have to carry the weight, it’s not “you guys aren’t worth anything and have to be below.” **They make you feel that you can, too, and they’re not going to look down on you in any way.**

For all of the participants, the discourses that denote an inequality or hierarchy between genders are unattractive. They understand that the fight against gender-based violence should be led by a movement that includes both men and women on an equal field. Cristina finds it inconceivable that there should be any type of inequality in the discourses against gender-based violence, either in the content or in the attitude of rage and aggressiveness. As we have stated, Luis was previously active in a men’s movement that he left because, among other reasons, the movement’s focus of analysis and action tended to centre on blaming all men for violence.

### Communicative Acts that Allow Liberty and the Establishment of an Egalitarian Dialogue

The third of the elements that all four participants agreed upon is how the discourses are structured. All of the participants agree about the necessity for a good argument in favour of what is being explained. Examples that help illustrate what is being explained should be added to the coherence demonstrated by the speakers. In their stories, the participants remember that in the past, they had constructed a narrative about gender-based violence different from the one that they now defend. Speaking with and getting to know other people (at lectures, workshops and more informal spaces) whose communicative acts transmitted what we have described in these results (coherence, unity between the languages of ethics and desire, and confidence when opposing any kind of violence) made the participants question themselves about their previous narratives.

Here, Luis tells us about the process of evolution that he has seen his students follow when they have participated in anti-violence workshops centred on the messages noted above. Luis chooses the metaphor of the lasso to exemplify how the workshop leader presents his arguments to the boys and girls, leaving them enough space and establishing an environment of trust so that they can give their opinions, are willing to present their own experiences, etc. As the leader gets to know the group and its situation better, he or she tightens the lasso, attempting to get to the point and showing students an alternative.

Luis: When a workshop leader, man or woman, who really responds to the egalitarian model. **the first thing the leader does is listen, not indoctrinate you.** Once you have listened, **you have to put yourself on an equal footing, explain, present an alternative model**.

In the workshop that my female students were in recently, what the guy did was like the rope, first he caught them but didn’t tighten, in the sense that he **lets them talk and gives them examples,** and when he sees that they begin to speak openly about the socialisation that they’ve had, what he does is tighten the cord to **begin to question them, like so**: ”is that the model you want to follow? These are the advantages of an alternative, egalitarian model. **I let you talk, tell me your opinions.** And we’re going to try to make it so you can evaluate, out of all of the options, which is the best so that you live with more equality.

**He succeeds in getting them not to close ranks, using an unassuming attitude, never losing his smile,** that’s important. **He says things clearly, he gives examples,** and as they start to realise: “hell, that’s true, that’s happened to me,” they begin to reflect and interact. And the **interaction amongst themselves** also helps them change their way of thinking. They reach a moment when they forget that they’re speaking with a researcher or workshop leader, and **they see him as an equal**, and they enter into a sincere dialogue, putting everything on the table to see which is the best option. It’s a reflection that goes on.

Cristina shares her own experience, specifically, how she has been modifying her own attractiveness models and constructing a new narrative about them for herself because of the interactions established in spaces of egalitarian dialogue. The example below centres on the visions that Cristina had both about the protagonist of the film “Coyote Ugly,” and about the film in general.

Cristina: This film, “Coyote Ugly,” the first time I saw it I thought “wow what a shitty movie.” Then, starting from that, I was talking with someone, **they talk to you with interest**, and you see it with new eyes.

What changed for me was how they talked to me about the guy. When they started to talk and say to me, “Look how the guy treats her,” to show me that “she’s better off…,” I saw the movie through different eyes. **They asked me those questions not with a categorical attitude, they were questions that are really far-reaching because they’re really normal**: “Let’s address actual things that everyone can understand.” With the dialogue you learn about how the guy is, it’s not “I’ll pass you a video and that’s it.”

For me **this dialogue is important, the tone in which it’s said, a more dialogic tone.** “After you can do what you want, but I’m presenting to you what there is and how it is. You can look with these eyes and analyse in this way, and afterward you live your life however you want to, but it’s important that you have this information.”

The fact that they state things clearly is definitely good to start changing your mind. However, also **you have to have a lot of space to talk about these issues and the different aspects they implicate.** It’s not just one thing that you say, “OK, I believe it and I will change.”

What also changes about the other discourse is **being very careful about what is said, how it’s said, and what I’m doing**. In other words, I am careful about how I present myself. That is, even behind a black screen, you can’t see the physical image of the person, the tone of voice, the argumentation they use. that helps. It’s the same if it’s someone who’s handsome or not, **if you present yourself in an attractive way that shows that you believe what you are saying, it reaches farther.**

Both of the participants and the adolescents about whom they spoke have been modifying their attractiveness models and constructing a new narrative for themselves based on the interactions established in spaces of egalitarian dialogue. This transformation has not been produced by the transmission of content via communicative acts that showed pretensions of power but instead by making valid arguments. Because of the establishment of this egalitarian dialogue, the participants were also able to present their doubts and contradictions. The positioning of the speaker was never intended to impose his or her opinion but instead to simply share scientific knowledge, leaving each person free to then draw his or her own conclusions. For the participants, clarity, argumentation and equality have been keys to assimilating new arguments about affective and sexual relations, attraction, or gender identities.

Cristina recognises that her lived socialisation has generally led to more visible traditional masculinity models of domination. However, alternative – egalitarian – masculinity models tended to pass her by unnoticed or even, in some cases, to seem boring. However, interaction with people who show desire for alternative models of masculinity made her question some of her assumptions, and she began to show interest in those alternative models. For Cristina, clarity, argumentation, and equality have been key to assimilating new arguments about affective and sexual relations, attraction, or gender identities.

Cristina, Daniela, Juan, and Luis provide examples of this ability to assimilate new arguments. Those examples are both personal and observed in the framework of different lectures and conferences, where they have seen the evolution of other people’s process of analysis and reflection.

## Discussion

In the fight against gender-based violence, there has been a tendency to see the presence of men as either an unnecessary interference or even another example of men’s oppression of women, their intent to control, women, etc. (De Koker et al., 2014). We know that this posture is not generalised and there are many mixed movements and that even without having activist men amongst them, activists count men as part of the solution. The four people who participated in the fieldwork agree with this outlook, and they start with the need for men and women to count on each other to transform society.

It is because of this that from the perspective of action via language, we should direct our interventions toward collective action, which develops a different narrative about affective and sexual relations (Lehrner and Allen, 2008; Salceda et al., 2020).

In the fight against gender-based violence, victims are currently urged to find help within the community – to tell someone – and the community is urged to facilitate this process by demonstrating a proactive attitude (Melgar et al., 2021). The campaigns with the best results are those that rely on the entire community’s participation to act before violence erupts, while simultaneously developing preventive educational actions (Coker et al., 2011). However, beyond knowing that we should rely on the entire community and the content that we should transmit, both professionals in this field and the general population ask themselves the same question as Rosa: How should those messages be transmitted?

As laid out in the development of the scientific literature, when we spoke about the analysis of gender-based violence from a linguistic perspective, we encountered a multivariable problem that transcends gender, in other words, where it is not solely gender that conditions the type of communication that either reproduces or overcomes the problem (Stone, 2005; Palomares, 2009). Moreover, not every type of communication sets off transformative actions (Clark, 1992; Klein, 2006).

Beginning with the concept of communicative acts instead of the concept of speech acts when analysing the articulation of discourses that can contribute to overcoming gender-based violence allows us to take a step further. In our study, these components have allowed Daniela, Cristina, Juan, and Luis to identify which are the elements in communicative acts that enable the construction of this narrative: the unification of the languages of ethics and desire, showing confidence and bravery in opposing violence and publicly positioning oneself in favour of victims and contributing clear and concrete arguments that exemplify the alternative, taking into account that these arguments should be made in the context of an egalitarian dialogue. For example, in no case was equality perceived only through speech acts that expressed a propositional context. Instead, the treatment of other people (either of respect and admiration, or of criticism and disrespect) or tones of voice (either enraged and recklessly destructive, or confident, convincing, and presenting alternatives) made the difference between a communication that reproduced violence against women and one that was transformative, along with the level of impact that the speakers had on the listeners.

Our results have contributed general knowledge about the communicative acts that contribute to overcoming gender-based violence. In future studies, it would be interesting to provide a more in-depth analysis of the aspects that comprise the communicative acts. This would entail identifying the non-verbal language, the social context, and the tacit knowledge present in those dialogues that contribute to transmitting the union of the languages of ethics and desire, showing confidence and bravery in opposing violence and publicly positioning oneself in favour of victims, and contributing clear and concrete arguments that exemplify alternatives.

## Data Availability Statement

The raw data supporting the conclusions of this article will be made available by the authors, without undue reservation.

## Ethics Statement

The studies involving human participants were reviewed and approved by Community of Researchers on Excellence for All (CREA). The patients/participants provided their written informed consent to participate in this study.

## Author Contributions

All authors have made substantial contribution to the article, and read and agree to the publication of the contents of the article. ED and PM contributed to the review of the scientific literature on communicative acts that overcome gender violence. ED, PM, SG-C, and GL contributed to the fieldwork and subsequent data analysis. ED reviewed and coordinated the final article as a whole.

## Conflict of Interest

The authors declare that the research was conducted in the absence of any commercial or financial relationships that could be construed as a potential conflict of interest.

## Footnotes

1 Office of the Dean (2003–2004). Tell Someone! Responding to Sexual Harassment, Sexual Assault and Rape. Harvard College. http://www.womensaid.org.uk/, https://alteristic.org/services/green-dot/

2 The characteristics of NAM have been collected in the state discussed in this article. For more information, please see this monograph’s introductory article.
